# Spectral Trajectory Analysis of Drug Release from Bioprinted Alginate-Based Hydrogels

**DOI:** 10.1021/acsomega.6c01437

**Published:** 2026-09-16

**Authors:** Antonio de Nigris, Luigi Ambrosone

**Affiliations:** Department of Medicine and Health Sciences, V. Tiberio, Molise University, Via De Sanctis, Campobasso 86100, Italy

## Abstract

The release of molecular probes from soft hydrogel matrices involves coupled transport and structural relaxation processes that are difficult to capture using predefined kinetic models. Here we investigate the release dynamics of indocyanine green from bioprinted alginate-based hydrogels, including alginate-gelatin and calcium-cross-linked alginate-poly­(vinyl alcohol) matrices, using a data-driven spectroscopic approach. Time-resolved UV–vis absorption spectra collected in the surrounding aqueous phase are analyzed by singular value decomposition to construct reduced-dimensional trajectories in spectral space. The spectral evolution exhibits a rapid initial increase followed by progressive saturation, indicating a transition from transient release to a quasi-stationary regime. Singular value analysis reveals an effectively low-dimensional dynamics, with two dominant modes capturing the essential temporal behavior across both systems. The resulting trajectories provide characteristic time scales and a compact, model-independent representation of release pathways directly from spectroscopic observables.

## Graphical abstract

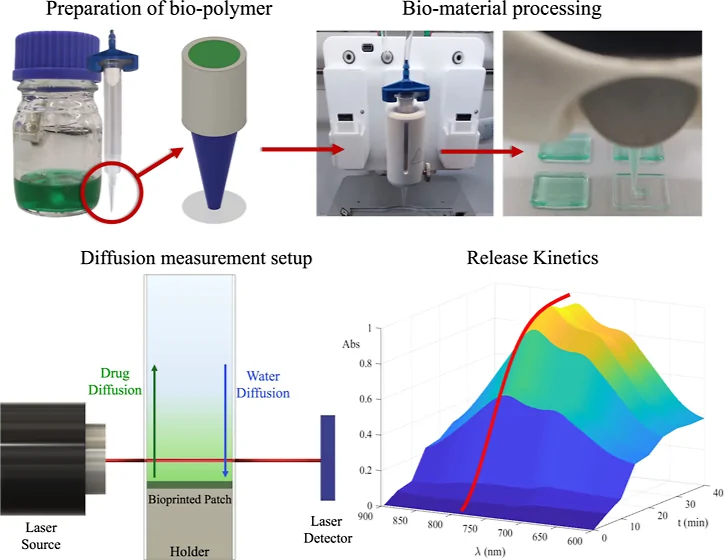

## 1 Introduction

Soft hydrogel-based materials are increasingly employed as platforms for controlled drug delivery, owing to their biocompatibility,^1^ tunable physicochemical properties,^2^ and structural similarity to biological tissues.^3^ In these systems, drug release typically results from the interplay of multiple concurrent processes, including polymer hydration, matrix swelling, solute diffusion, erosion, and interfacial transport.^4^ As a consequence, the spatiotemporal dynamics of release are often highly nonlinear and difficult to disentangle experimentally.^5^ Despite this intrinsic complexity, drug release kinetics are frequently interpreted using simplified diffusion-based or empirical models,^6^ which assume homogeneous matrices, stationary transport coefficients, and well-defined release mechanisms. While such approaches can provide useful effective parameters, they often obscure the underlying dynamics and may fail when applied to supramolecular or multicomponent hydrogels, where structural reorganization, phase heterogeneity, and solvent-mediated transformations occur on comparable time scales.^7^ An additional challenge arises from the fact that many experimental techniques probe the system indirectly, typically through spectroscopic signals collected at fixed spatial locations in the surrounding medium. These measurements do not provide direct access to the full concentration field but instead reflect the local appearance and stabilization of the drug at the observation point.^8^ From a practical standpoint, this local signal is of primary relevance, as it corresponds to the time scale over which the drug becomes bioavailable in the vicinity of the delivery site. However, linking such local measurements to the underlying release dynamics remains a nontrivial task. Time-resolved optical spectroscopy offers a powerful, noninvasive means to monitor drug release in situ, but the interpretation of the resulting spectral data poses its own challenges.^9^ The recorded spectra often reflect the superposition of multiple evolving states of the drug and its environment, convoluted with transport processes occurring both within the hydrogel and in the surrounding fluid. Modern hydrogel-based delivery platforms often exhibit swelling, structural relaxation, heterogeneous transport pathways, and time-dependent variations in diffusivity. Under these conditions, classical analytical descriptions such as the Higuchi^10^ and Korsmeyer–Peppas^11^ models become inadequate, as they rely on restrictive assumptions—including rigid matrices, constant diffusion coefficients, and purely Fickian transport—that are not fulfilled in soft, dynamically evolving bioprinted systems. To overcome these limitations, data-driven strategies based on the analysis of time-resolved spectroscopic signals offer a powerful alternative.^12–14^ In particular, the combination of singular value decomposition with spectral trajectory mapping provides a model-independent framework capable of extracting intrinsic dynamical features directly from experimental data. By representing release dynamics as low-dimensional trajectories in a reduced spectral space, this approach captures the global organization of swelling, transport, and relaxation processes without imposing predefined kinetic laws. A key advantage of this trajectory-based perspective is that it yields physically interpretable descriptors—such as characteristic times, early time burst signatures, and late-stage network resistance—that remain valid even when the underlying mechanisms are complex or coupled. This makes the method especially valuable for the quantitative characterization and comparative assessment of drug release in advanced bioprinted hydrogel systems. In this work, we address these challenges by introducing a data-driven framework that maps time-resolved UV–vis absorbance spectra onto a low-dimensional spectral state space using singular value decomposition^15^ (SVD). Rather than fitting predefined kinetic or diffusion models, our approach identifies the intrinsic dynamical degrees of freedom directly from experimental data, providing a compact and physically meaningful representation of the release process. In this reduced space, the evolution of the system is naturally described as a trajectory, whose geometry encodes the coupled effects of transport, swelling, and release. We apply this methodology to bioprinted alginate-based hydrogel patches loaded with indocyanine green^6^ chosen as a spectroscopically active model compound. Bioprinted hydrogels span a wide spectrum of mechanical and transport behaviors, ranging from soft, weakly structured matrices to compact, cross-linked networks with pronounced relaxation dynamics. In this work, we consider two representative systems—non-cross-linked alginate-gelatin and ionically cross-linked alginate-poly­(vinyl alcohol)—loaded with the same spectroscopic probe. Although not intended as universal archetypes, these materials capture two limiting cases that are emblematic of distinct classes of hydrogel architectures: a highly swellable, diffusion-dominated matrix and a structurally constrained, relaxation-controlled network. This contrast provides an ideal testbed for evaluating model-independent analytical strategies. In particular, it highlights the need for data-driven approaches capable of resolving complex, coupled release mechanisms that fall outside the validity domain of classical models such as Higuchi^10^ and Korsmeyer-Peppas.^11,16^

## 2 Materials and Methods

### 2.1 Materials

Sodium alginate (Alg) was purchased from Farmalabor, with a pH between 6.00 and 8.00. Indocyanine green (ICG) was a medical device purchased from Diagnostic Green. Porcine gelatin (Gel, 300 bloom, Type A), poly­(vinyl alcohol) (PVA), calcium chloride (CaCl_2_) were purchased from Merck KGaA. All chemicals were used without any further purification and all solutions were prepared using ultra pure water (UPW) obtained from the Milli-Q purifier system.

### 2.2 Hydrogel Preparation and Bioprinting

Alg/Gel hydrogels (25.0 mL) were prepared by physically blending two separately prepared aqueous solutions, following a previously reported protocol.^17^ The alginate solution was obtained by dissolving 1.50 g of alginate powder in 12.5 mL of UPW preheated to 80 °C under vigorous magnetic stirring (500 rpm) for 1 h. In parallel, 0.750 g of gelatin powder was dissolved in 12.5 mL of UPW at 60 °C under continuous stirring for 1 h. The two solutions were combined at 80 °C and mixed for 15 min, followed by further homogenization for 90 s. The resulting Alg/Gel hydrogel was allowed to cool to room temperature and stored at 4 °C overnight prior to use. Alg/Gel/ICG hydrogels (25.0 mL) were prepared by adding 0.500 mL of an aqueous indocyanine green (ICG) solution (5.00 g L^–1^) to 24.5 mL of the Alg/Gel hydrogel base after cooling to room temperature, in order to minimize dye degradation. The mixture was gently stirred until homogeneous.

Alg/PVA hydrogels were prepared analogously.^18^ Alginate powder (0.713 g) was dissolved in 12.5 g of UPW preheated to 80 °C under magnetic stirring (500 rpm) for 45 min, yielding a 5.39 wt % solution. Separately, a 3.66 wt % PVA aqueous solution was prepared by dissolving 0.475 g of PVA in 12.5 g of UPW at 90 °C under gentle stirring (125 rpm) for 45 min. The two solutions were then combined and mixed at 80 °C under vigorous stirring (500 rpm) for 15 min. Alg/PVA/ICG hydrogels were obtained by adding 0.500 mL of ICG aqueous solution (5.00 g L^–1^) to the Alg/PVA mixture after cooling to room temperature, followed by stirring for 15 min. Hydrogel patches were designed as parallelepiped constructs with lateral dimensions of 10.0 mm × 10.0 mm and a thickness of 0.400 mm using Autodesk Inventor. All patches were fabricated by extrusion-based bioprinting using a BIO X bioprinter (CELLINK, Twin Helix). For each print, 2.50 mL of hydrogel precursor solution was loaded into a 3.00 mL printing cartridge and degassed in an ultrasonic bath for 15 min to remove entrapped air bubbles. Bioprinting was performed in triplicate at a printing temperature of 39.0 °C for Alg/Gel and 25.0 °C for Alg/PVA hydrogels, using an applied pressure of 100 or 30 kPa respectively, a deposition speed of 5.00 mm s^–1^, an infill density of 40% and a 27-gauge nozzle.

### 2.3 Optical Measurement Setup and Data Acquisition

The optical setup employed to monitor the release of ICG from the bioprinted patches is schematically illustrated in Figure 1. The system was designed to probe, in a time-resolved and noninvasive manner, the local optical response of the aqueous phase in proximity to the releasing patch. A monochromatic laser source irradiated a standard spectrophotometric cuvette containing the dried patch. The patch was mechanically supported by a custom-fabricated silicone parallelepiped, which ensured a fixed and reproducible positioning within the cuvette. This spacer defined a constant vertical distance between the patch surface and the optical axis, allowing the probing beam to intersect the aqueous phase at approximately 1 mm above the patch. Upon addition of ultrapure water to the cuvette, the patch was brought into contact with the solvent, initiating hydration, swelling, and subsequent release of ICG. The optical beam traversed the water bath parallel to the patch surface, intercepting the region where the dye progressively accumulated as a result of diffusion and convective transport. The transmitted light was collected by a photodetector and digitized by a dedicated acquisition system, generating time-resolved absorption spectra that were stored for subsequent analysis. This measurement geometry ensured that the optical signal originated from a fixed spatial location throughout the experiment. As a consequence, the recorded spectra represent a localized probe of the temporal evolution of the dye concentration in the surrounding aqueous bath, rather than a spatial average over the entire volume. The combined effects of matrix swelling, dye release, diffusion, and convection are therefore encoded in the temporal evolution of the measured spectral data.

**Figure 1:**
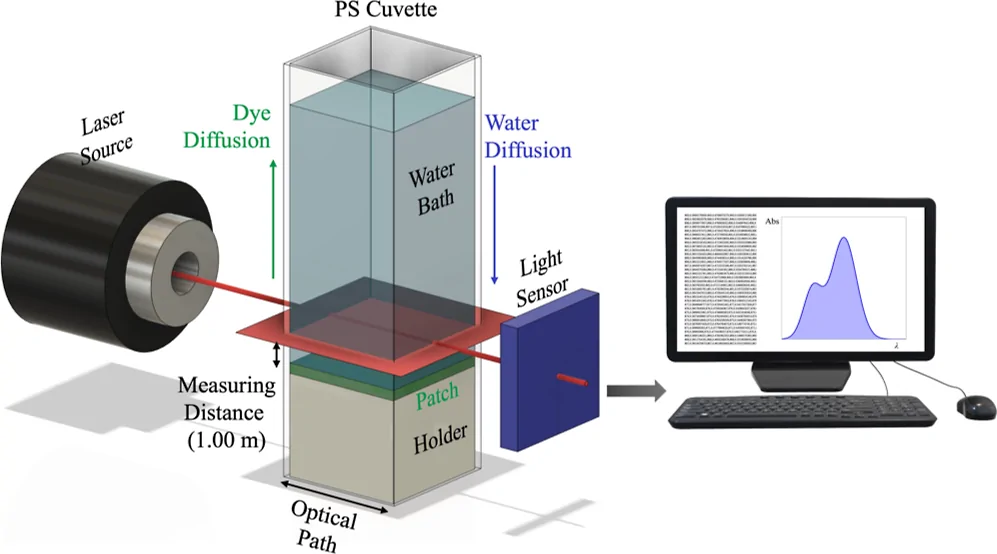
Scheme of the optical setup used to monitor ICG release from bioprinted Alg/Gel patches. The dried patch is placed on a silicone support inside a spectrophotometric cuvette to maintain a fixed distance from the optical axis. Upon addition of ultrapure water, hydration and dye release are initiated. A laser beam probes the aqueous phase approximately 1 mm above the patch surface, and the transmitted light is detected and processed to obtain time-resolved absorption spectra.

## 3 Theoretical Aspects

### 3.1 Theoretical Framework and Data Analysis

Let *C*(**r**, *t*) be the dye concentration at position **r** at time *t*. The transport of ICG is governed by a generalized advection-diffusion equation^19^ of the form

1 $$\frac{\partial C}{\partial t}=D\nabla^{2}C-\nabla \cdot \left(vC\right)+S\left(r,t\right)$$

where *D* is the effective diffusion coefficient, **v** is the velocity field accounting for convective transport, and *S*(**r**, *t*) represents a source term associated with dye release from the patch. This term implicitly incorporates the complex interplay between swelling, matrix relaxation, and erosion processes.

Importantly, the spectrophotometric measurement does not directly access the spatially resolved concentration field *C*(**r**, *t*), but instead records an optical absorbance spectrum *A*(λ, *t*), which represents a spatially weighted integral of the concentration field.^20^ As a first approximation, the measured absorbance can be written as

2 $$A\left(\lambda ,t\right)=\int_{V}\epsilon \left(\lambda \right)C\left(r,t\right)W\left(r\right)dr$$

where ϵ­(λ) is the molar extinction coefficient of ICG and *W*(**r**) is a weighting function that incorporates the geometry of the cuvette and the optical collection volume.

In the present experimental configuration, the optical axis intersects a fixed and well-defined region of the aqueous bath throughout the entire measurement. Consequently, the weighting function can be approximated as

3 $$W\left(r\right)≃\delta \left(r-r_{0}\right)$$

where *r*_0_ is a time-invariant spatial position and δ denotes the Dirac delta-distribution.^21^ Under this approximation, eq 2 reduces to

4 $$A\left(\lambda ,t\right)=\epsilon \left(\lambda \right)C\left(r_{0},t\right)l$$

where l is the effective optical path.

Each recorded spectrum can therefore be interpreted as a spectral snapshot of the temporal evolution of the dye concentration within a fixed observation volume of the aqueous phase.^22^ If the system were characterized by a single chemical species and a unique conformational state, the resulting data matrix would be of rank one.

The experimental spectra are acquired at discrete times *t*_1_, *t*_2_,···, *t*_*N*_ and sampled over a wavelength grid λ_1_, λ_2_,···, λ_*M*_ yielding a data matrix which inherently encodes the coexistence of multiple spectral states of ICG, as well as the temporal evolution of the release process. The SVD of this matrix

$$A=USV^{T}$$

provides a model-independent factorization into three components: *U* = [*u*_1_(λ), *u*_1_(λ),···], whose columns represent orthonormal spectral modes; *S* = diag­(σ_1_, σ_2_,···) whose elements quantify the relative of each mode; and the matrix *V* = [*v*_1_(*t*), *v*_2_(*t*),···], whose columns describe the temporal evolution of the corresponding spectral components. Together, these quantities define a reduced representation of the release dynamics as a trajectory in a low-dimensional spectral state space.

This decomposition allows each experimental spectrum to be expressed as a linear combination of orthonormal spectral-temporal modes

5 $$A\left(\lambda ,t\right)=\sum_{k=1}^{r}\sigma_{k}u_{k}\left(\lambda \right)v_{k}\left(t\right)$$

where *r* is the numerical rank of the data matrix. The functions *u*_*k*_(λ) represent characteristic spectral signatures associated with distinct average states of the system, while the coefficients *v*_*k*_(*t*) describe the temporal evolution of these states.

It is important to emphasize that the SVD does not impose any a priori diffusion, kinetic, or transport model. Rather, it constructs an optimal, data-driven basis that captures the dominant correlations present in the experimental data set.^23^

In many experimentally relevant cases, the singular values exhibit a clear hierarchical separation

6 $$\sigma_{1}>\sigma_{2}\gg \sigma_{3},\cdot \cdot \cdot$$

indicating that the essential dynamics of the system can be faithfully represented within a low-dimensional subspace. Under this condition, the absorbance can be accurately approximated by retaining only the first two modes

7 $$A\left(\lambda ,t\right)=\sigma_{1}u_{1}\left(\lambda \right)v_{1}\left(t\right)+\sigma_{2}u_{2}\left(\lambda \right)v_{2}\left(t\right)$$

At each time point, the system is therefore represented by a vector of reduced coordinates

8 $$x\left(t\right)=\left(v_{1}\left(t\right),v_{2}\left(t\right)\right)$$

which defines a trajectory in a reduced two-dimensional space

9 $$\Gamma =\left\{x\left(t\right)\in R^{2}\right\}$$

In contrast, the full concentration field evolves in an infinite-dimensional functional space

10 c(r,t)∈H

where H denotes an appropriate Hilbert space of admissible concentration fields.^24^ The SVD therefore defines a projection map

11 Φ:c(r,t)→x(t)

which embeds the high-dimensional transport dynamics into a low-dimensional spectral state space.

12 $$\frac{dc\left(r_{0},t\right)}{dt}=D\nabla^{2}c\left|r_{0}-\nabla \cdot \left(vc\right)\right|r_{0}+S\left(r_{0},t\right)$$

The experimentally observed trajectory **x**(*t*) thus represents the image of the underlying transport and release dynamics projected onto the dominant spectral modes. At the fixed observation point, the local concentration obeys

13 $$c\left(r_{0},t\right)\to x\left(t\right)$$

which can be formally described by an effective dynamical system

14 $$\frac{dx}{dt}=F\left(x,t\right)$$

In other words, the method reconstructs the projection of the transport dynamics rather than the full concentration field. The experimentally measured signal can thus be written as

15 $$A\left(\lambda ,t\right)=c_{free}\left(r_{0},t\right)\epsilon \left(\lambda ,state\right)$$

where *c*_free_ is the concentration of optically active (free) ICG at the measurement point and ϵ­(λ, state) accounts for possible spectral variations associated with different molecular or supramolecular states. Overall, the process can be summarized schematically as

16 $$Release\overset{Diffusion/Convection}{\to }Local\;Bioavailability$$

where the measurement directly probes the time-dependent appearance and stabilization of the drug at a fixed location in the aqueous bath.

The theoretical framework introduced above establishes a direct connection between the local transport dynamics of ICG and the evolution of time-resolved optical spectra recorded at a fixed observation point. Within this perspective, the measured absorbance matrix represents a low-dimensional projection of a much higher-dimensional transport process, whose dominant features can be systematically extracted through singular value decomposition and analyzed in terms of reduced spectral trajectories.

## 4 Results and Discussion

### 4.1 Analysis of Non-Crosslinked Alg/Gel/ICG Patch

We now turn to the experimental results. We first present the raw UV–vis absorption spectra acquired during the release of indocyanine green from the Alg/Gel patch into the aqueous bath. These spectra constitute the primary experimental observable and provide a qualitative picture of the release process, upon which the subsequent SVD-based analysis and reduced-state interpretation are built. Figure 2 shows the time-resolved UV–vis absorption spectra recorded during the release of ICG from the bioprinted Alg/Gel patch into pure water. At early times, the absorbance increases rapidly over the entire spectral range, indicating the prompt appearance of the dye in the probed region of the aqueous bath. As time progresses, the rate of absorbance increase gradually decreases, and successive spectra tend to accumulate, suggesting the approach toward a quasi-stationary concentration regime at the measurement point. Notably, the spectral shape remains essentially unchanged during the experiment, suggesting that the dominant absorbing species is preserved over time and that no major chemical degradation or spectral transformation of ICG occurs within the observation window. Therefore, the temporal evolution of the spectra primarily reflects changes in local concentration rather than changes in molecular identity.

**Figure 2:**
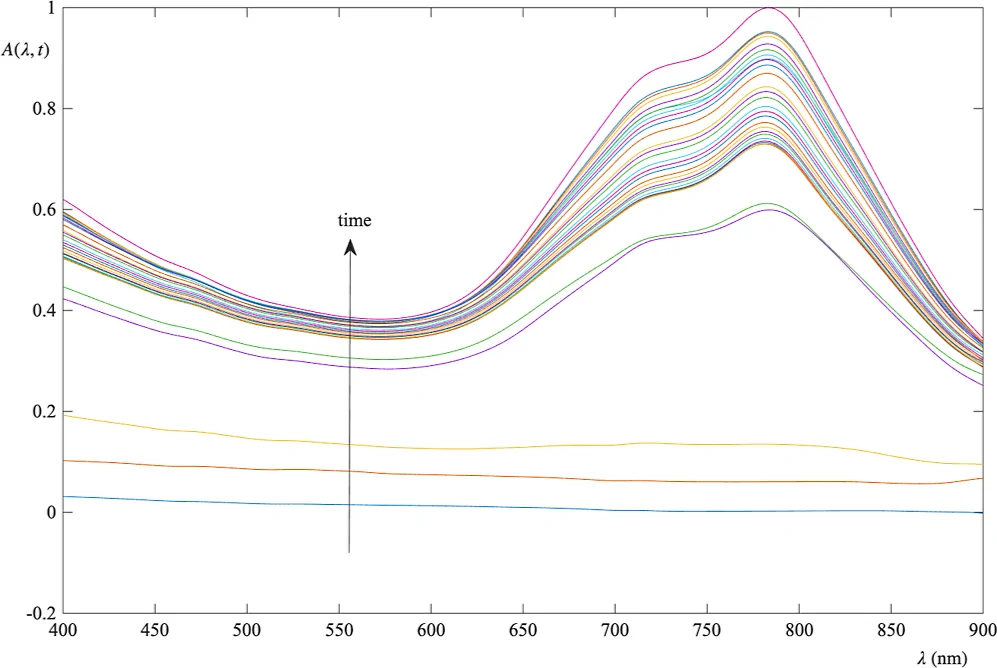
Time-resolved UV–vis absorption spectra of ICG released from Alg/Gel patch into the aqueous bath, recorded at a fixed distance from the optical axis.

The SVD of the absorbance matrix is shown in Figure 3 as a function of the component index. It spans approximately 4 orders of magnitude before reaching a plateau, demonstrating that the release dynamics are effectively low-dimensional. The smallest singular value was excluded from the plot, as it was 8 orders of magnitude smaller than the preceding components and dominated by numerical noise. Consequently, only the first two singular components are retained for further analysis, as they capture the essential structure of the spectral evolution while filtering out noise-dominated contributions.

**Figure 3:**
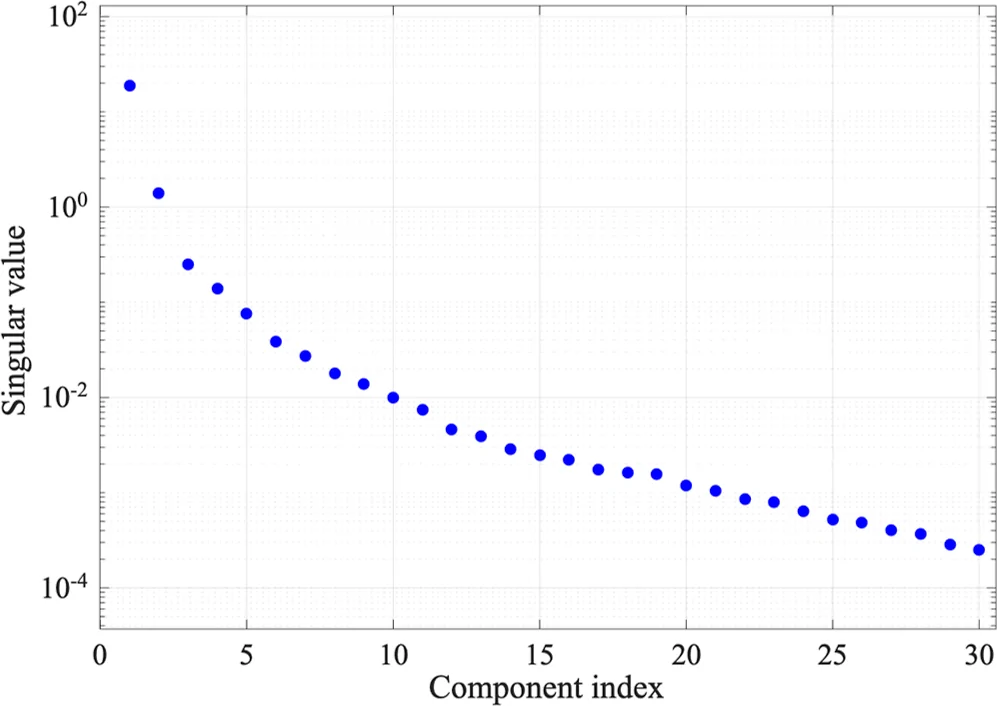
Singular values from the SVD of the centered absorbance matrix on a logarithmic scale. The rapid decay indicates that the dynamics are governed by a small number of dominant modes.

The combined dynamics of the system are most clearly visualized in the *V*_1_ – *V*_2_ phase portrait shown in Figure 4. The trajectory initially rises steeply, indicating a rapid excursion in reduced space associated with the early time release burst. This is followed by an extended region where the trajectory remains close to its maximum over a broad range of points, corresponding to a prolonged quasi-steady regime in which the local concentration evolves slowly. Only at the final stages of the experiment do the last two points deviate from this plateau and move toward lower values, signaling the approach to a terminal equilibration regime. The broad maximum observed in the trajectory suggests that the system spends a significant fraction of the experimental time in a dynamically constrained state, where release, diffusion, and dilution are effectively balanced. Importantly, the smooth and continuous nature of the trajectory indicates that the release process proceeds without abrupt transitions or instabilities, supporting a diffusion-controlled mechanism modulated by hydrogel swelling and matrix relaxation. Taken together, the spectral evolution, singular-value hierarchy, temporal modes, and reduced-space trajectory provide a coherent picture of ICG release from the Alg/Gel patch. The process is characterized by an initial fast release component, followed by a slower, diffusion-dominated regime that leads to stabilization of the local dye concentration at the measurement point. Rather than reconstructing the full concentration field, the SVD-based approach captures the projection of the underlying transport dynamics onto a low-dimensional observable space. The resulting trajectories thus encode the essential temporal features of drug availability in the surrounding medium. This framework provides a robust, model-free methodology to extract characteristic release times and dynamical regimes directly from spectroscopic data, with potential applicability to a wide range of hydrogel-based drug delivery systems.

**Figure 4:**
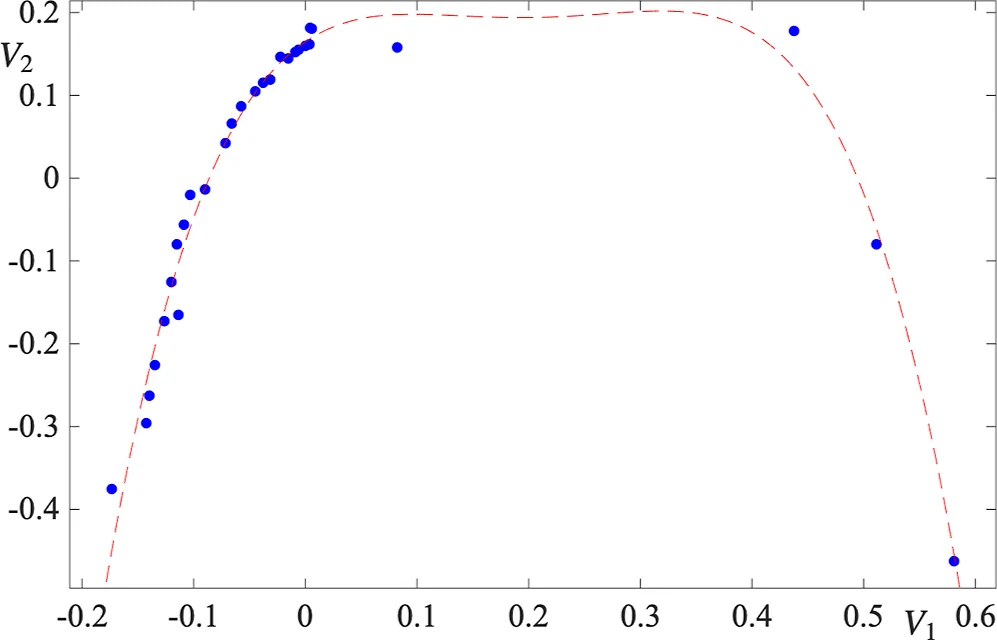
Trajectory in the reduced state space (*V*_2_ vs *V*_1_) for the Alg/Gel/ICG system, showing a rapid increase followed by a broad maximum and a slow decay toward the final state (dotted line is a guide for the eyes).

### 4.2 Analysis of Crosslinked Alg/PVA/ICG Patch

Figure 5 shows the time-resolved UV–vis absorbance spectra of indocyanine green released from the cross-linked Alg/PVA patch. The spectral profiles closely resemble those observed for the non-cross-linked Alg/Gel system, indicating that the optical signature of ICG remains unchanged by the different polymeric matrix and cross-linking chemistry. No additional absorption features or spectral distortions are detected, confirming that the release process does not induce aggregation or chemical modification of the dye. Compared to the Alg/Gel patch, the appearance of the characteristic ICG absorption bands occurs at earlier times, anticipating the overall release dynamics. This temporal shift is consistent with the significantly reduced characteristic times extracted from the SVD-based analysis, and points to a faster establishment of local dye availability in the surrounding aqueous phase. While the Alg/PVA network is structurally more compact due to calcium cross-linking, the earlier spectral onset suggests that surface-mediated transport and enhanced convective exchange dominate the early stage dynamics, overcoming the increased network density. These observations highlight that, although the spectral evolution follows the same qualitative pathway in both systems, the time scale at which the system explores the spectral state space is strongly modulated by the physicochemical properties of the hydrogel. In this sense, the experimental spectra provide the first indication of accelerated release kinetics in the cross-linked patch, later quantified and rationalized through the reduced SVD representation and spectral trajectory analysis. In the cross-linked Alg/PVA/ICG system, the singular value spectrum exhibits a markedly different structure compared to the non-cross-linked Alg/Gel/ICG patch (Figure 6). While the singular values still decrease monotonically with increasing index, the separation between the leading components is less pronounced. Specifically, several singular values lie within the same order of magnitude before a plateau is reached, followed by a sharp drop of the smallest components.

**Figure 5:**
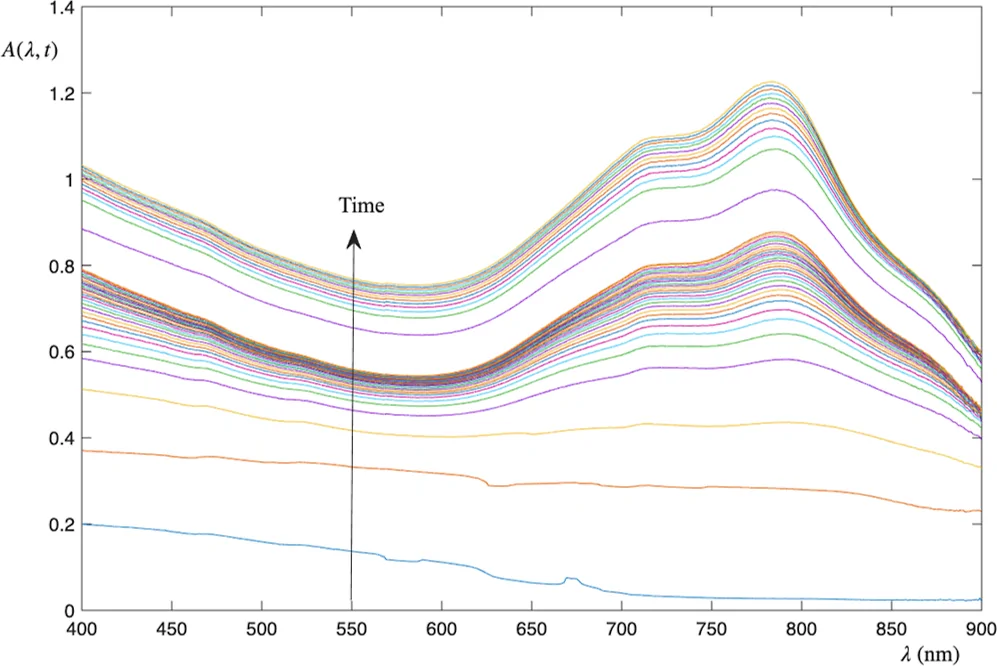
Time-resolved UV–vis absorbance spectra of indocyanine green released from the cross-linked Alg/PVA patch into an aqueous bath, at a fixed distance from the optical axis.

**Figure 6:**
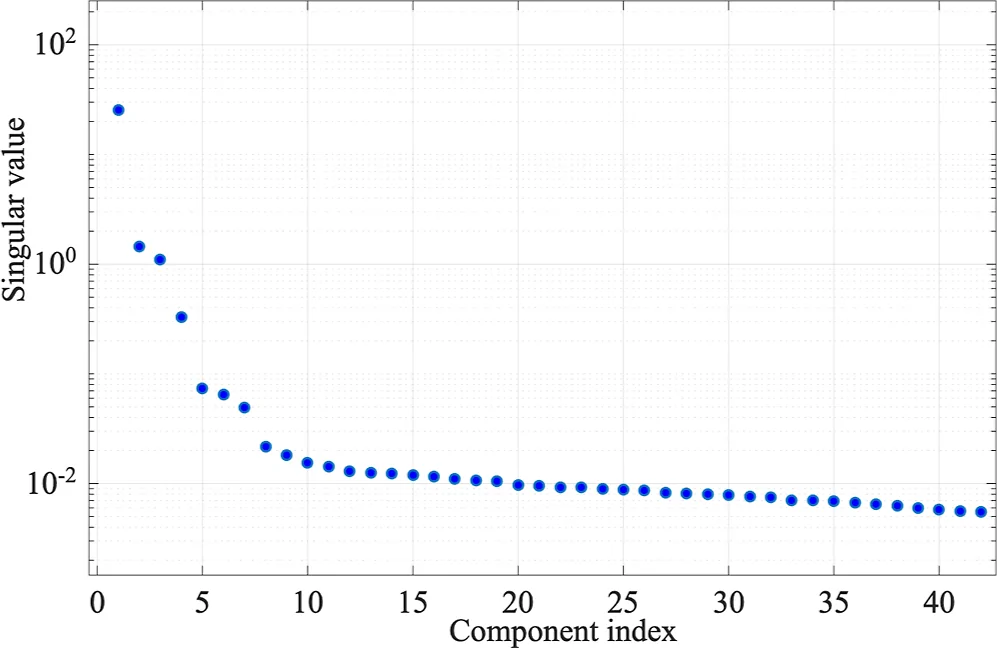
Singular values from the SVD of the centered absorbance matrix, shown as a function of component index on a logarithmic scale.

This behavior indicates that the release dynamics cannot be captured by only one or two dominant modes, but rather involves a larger number of coupled dynamical degrees of freedom. Physically, this reflects the increased structural complexity of the cross-linked network, where dye release is not governed solely by simple diffusion, but is mediated by additional processes such as network relaxation, partial disruption of ionic cross-links, and heterogeneous transport pathways. Importantly, the absence of a strong singular value gap does not represent a limitation of the method. On the contrary, it demonstrates the sensitivity of the SVD approach to changes in the underlying physicochemical mechanisms, allowing the intrinsic complexity of the system to emerge directly from the data without imposing any predefined kinetic model. The reduced spectral trajectory in the (*V*_1_, *V*_2_) space for the cross-linked Alg/PVA/ICG (Figure 7) patch displays a distinctive morphology, markedly different from that observed in the non-cross-linked system. In this case, *V*_2_ initially decreases, reaches a well-defined horizontal inflection, then continues toward a deep minimum before eventually rising toward a late-time plateau. This nonmonotonic trajectory reveals the presence of multiple sequential dynamical regimes. The initial descent is associated with early stage transport and surface release, while the extended inflection region suggests a transient balance between dye liberation and structural constraints imposed by the cross-linked polymer network. The deep minimum indicates a delayed release phase, likely controlled by the progressive loosening or partial rupture of Ca^2+^-mediated junctions, followed by a final regime characterized by enhanced transport and accumulation in the surrounding bath.

**Figure 7:**
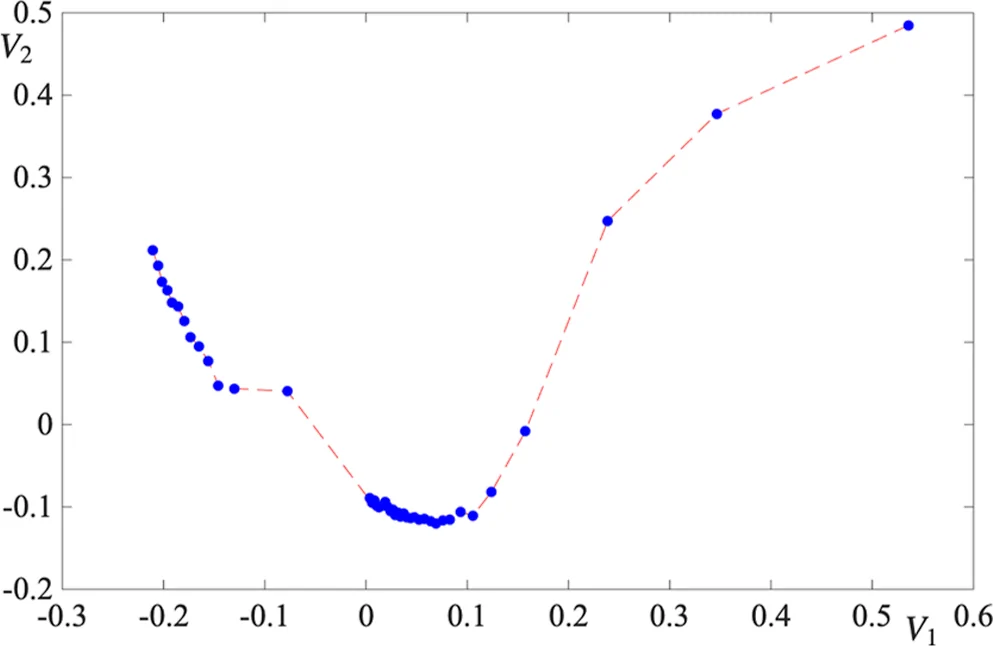
*V*_2_ – *V*_1_ trajectory for the Alg/PVA/ICG system, showing a multiregime release dynamics (dotted line is a guide for the eyes).

### 4.3 Characteristic Release Times and Local Bioavailability

To quantitatively characterize the release dynamics at the measurement point, two characteristic times are generally introduced, i.e. *t*_50_ and *t*_90_, defined as the times required for the experimental signal to reach 50 and 90% of its asymptotic value, respectively. However, in the present analysis, the extract characteristic times do not represent the cumulative fraction of released drug. Rather, they quantify the time scale over which dye becomes detectable and stabilizes locally at the fixed measurement position (Figure 1). Specifically, the analysis focused on the cumulative contribution of the dominant temporal modes, which capture the physically relevant dynamics of the system while efficiently filtering high-frequency experimental noise. The leading reduced temporal signal was defined as

17 $$s_{1}\left(t\right)=U_{1}\left(t\right)\sigma_{1}$$

where σ_1_ is the largest singular value and *U*_1_ is the corresponding temporal coordinate. This quantity represents the dominant contribution to the time-dependent optical signal measured at the fixed observation point and serves as a robust proxy for the local concentration dynamics of ICG in the surrounding aqueous phase. An onset time *t*_onset_ was defined as the time at which the signal reaches a small fraction α of its maximum value

18 $$s_{1}\left(t_{onset}\right)=\alpha s_{1}^{\max}$$

with α ≪ 1. The time of semi-availability was then defined as

19 $$s_{1}\left(t_{50}\right)=0.5s_{1}^{\max}$$

while the saturation time was identified as the time beyond which further variations of the signal become negligible, according to

20 $$\left|\frac{ds_{1}}{dt}\right|\leq \varepsilon$$

where ε is a small threshold accounting for experimental noise.

For the non-cross-linked Alg/Gel/ICG patch, this procedure yielded *t*_50_ = 75.5 min, *t*_90_ = 134.4 min, while the calcium-cross-linked Alg/PVA/ICG patch exhibited *t*_50_ = 25.4 min, *t*_90_ = 83.1 min. Such results indicate a markedly faster establishment of local drug availability in the cross-linked system, despite its more compact polymeric network. This behavior can be rationalized by examining the reduced SVD representation, which reveals how differences in early- and midtime dynamics arise from measurable features of the dominant spectral mode rather than from network density alone. In particular, polymer–water affinity, swelling kinetics, and surface-mediated transport manifest directly in the geometry and timing of the reduced trajectories. Indeed, the early time growth rate of *s*_1_(*t*), together with the steep initial displacement of the (*V*_1_, *V*_2_) trajectory, define a burst index. In the non-cross-linked Alg/Gel patch, the initial excursion spans approximately Δ*V*_1_ ≈ 0.55 and Δ*V*_2_ ≈ 0.30, accounting for nearly 40% of the total arc length. In contrast, the cross-linked Alg/PVA patch exhibits a much smaller initial displacement (Δ*V*_1_ ≈ 0.20, Δ*V*_2_ ≈ 0.10), corresponding to only 10–15% of the total arc length. The swelling index, defined by the duration of the high-velocity region or equivalently by the width of the inflection in *s*_1_(*t*), also differs markedly between the two systems. The Alg/Gel patch maintains elevated trajectory velocity over 60–70% of the experiment, indicating a broad swelling-controlled regime. Conversely, the Alg/PVA patch reaches its velocity maximum within the first 15–20% of the experiment, yielding a much narrower swelling window. The network resistance index, quantified by the residual arc length and curvature in the late portion of the trajectory, is low in the Alg/Gel patch (residual arc length <5%), but significantly higher in the Alg/PVA system, where 20–25% of the total arc length accumulates at late times and the trajectory exhibits pronounced curvature. These signatures reflect delayed transport associated with the relaxation of Ca^2+^-mediated junctions and the progressive reorganization of the polymer network.

The characteristic times also admit a direct geometrical interpretation within the reduced spectral state-space representation, fully consistent with the quantitative descriptors extracted above. At early times, the rapid displacement of the trajectory away from the origin reflects the swift appearance of indocyanine green at the measurement point, corresponding to the steep initial growth of *s*_1_(*t*) and to the large early arc-length fraction observed in the Alg/Gel patch. The time *t*_50_ is reached while the trajectory is still evolving along the steep portion of its path, where transport- and swelling-driven processes dominate and small temporal variations correspond to large displacements in spectral space. As the trajectory approaches its broad maximum or inflection region, the temporal evolution progressively slows and the reduced coordinates change more gradually. This geometrical stabilization coincides with the approach to *t*_90_ and is consistent with the markedly different network resistance indices of the two systems.

### 4.4 Synthesis and Extraction of Characteristic Release Times

Classical analytical models such as Higuchi or Korsmeyer-Peppas cannot be applied globally to the present systems. Both models assume a rigid, nonswelling matrix, constant diffusivity, and purely Fickian transport, whereas the hydrogels studied here exhibit swelling, time-dependent structural relaxation, and non-negligible convective contributions. The Higuchi equation additionally requires uniform initial drug distribution and sink conditions.^10^ Similarly, the Korsmeyer-Peppas model is an empirical early time approximation valid only for the initial fraction of release (typically below 60%), and assumes an approximately constant geometry and a single dominant mechanism.^11,16^

Although the assumptions underlying the Higuchi and Korsmeyer-Peppas models are not strictly satisfied in these swelling and structurally evolving hydrogels, it is nevertheless informative to compare the characteristic times extracted from these classical early time approximations with those obtained from spectral integration and SVD analysis. Such a comparison highlights the extent to which diffusion-based models capture only the initial portion of the release process and quantifies their deviation from the global dynamical behavior. To quantitatively compare the release behavior of the two hydrogel systems, three complementary descriptors were extracted from the experimental and model-based analyses. First, the UV–vis spectra were integrated over the 560–900 nm range to obtain a scalar observable *A*(*t*) proportional to the local concentration of released dye. The resulting curve was normalized as *M*_*t*_/*M*_∞_ = *A*(*t*)/*A*(*t*_end_), where *A*(*t*_end_) was estimated from the average of the final measurement points to mitigate baseline drift. The time at which this normalized signal reached 0.5 was taken as the direct experimental estimate $t_{50}^{Area}$ . Second, the dominant temporal mode obtained from the SVD decomposition of the full spectral data set provided a global descriptor of the release dynamics. The corresponding semi-availability time $t_{50}^{SVD}$ was defined as the time at which the leading SVD coefficient reached half of its asymptotic value, capturing the overall progression of the release process beyond the local measurement point. Finally, early time kinetic fits were performed using the Higuchi model, *M*_*t*_/*M*_∞_ = *k*_*H*_ *t* ^1/2^, applied only to the initial portion of the normalized curve (*M*_*t*_/*M*_∞_ < 0.6). The fitted parameter *k*_*H*_ yielded the analytical estimate $t_{50}^{\left(H\right)}$ = (0.5/*k*_H_)^2^ Korsmeyer-Peppas fits did not converge for either system, indicating the absence of a stable power-law regime. Together, these three metrics provide a coherent multiscale quantification of release kinetics, linking local spectral evolution, global dynamical structure, and classical diffusion-based approximations. The characteristic times reported in Table 1 highlight the complementary nature of the three extraction methods. For the non-cross-linked Alg/Gel patch, the area-based estimate $t_{50}^{Area}$ = 12.25 min reflects the rapid initial appearance of dye at the measurement point, whereas the SVD-based value $t_{50}^{SVD}$ = 75.5 min reveals that a substantial portion of the global dynamical evolution occurs well beyond this early rise. The Higuchi estimate $t_{50}^{\left(H\right)}$ = 49.9 min lies between these two values but still underestimates the global time scale by approximately 34%, consistent with the extended swelling-controlled regime captured by the reduced trajectories. For the cross-linked Alg/PVA patch, the discrepancy is even more pronounced. The local signal reaches half of its asymptotic value extremely rapidly ( $t_{50}^{Area}$ = 2.50 min), yet the SVD analysis indicates that the full release dynamics unfold over a much longer time scale ( $t_{50}^{SVD}$ = 25.4 min), reflecting the relaxation of the Ca^2+^-mediated network. The Higuchi estimate $t_{50}^{\left(H\right)}$ = 3.28 min captures only the very early diffusion-like regime and underestimates the global time scale by nearly 90%. The failure of Korsmeyer-Peppas fits to converge for either system further confirms that no single power-law regime dominates the release process. Overall, these results demonstrate that classical early time models provide only partial information, whereas the SVD-based framework consistently resolves both fast and slow dynamical components of the release process.

**Table 1:** Characteristic Release Times Extracted from Spectral Integration, SVD Analysis, and Higuchi Fitting

system	$t_{50}^{Area}$ (min)	$t_{50}^{SVD}$ (min)	$t_{50}^{\left(H\right)}$ (min)
non-crosslinked Alg/Gel	12.25	75.5	49.9
crosslinked Alg/PVA	2.50	25.4	3.28

It is worth noting that the present analysis focuses on ICG as a single model probe, however the methodology developed here is not limited to this specific molecule. The SVD-based trajectory framework only requires that the transported species exhibit a stable and distinguishable optical signature over the experimental time scale, a condition satisfied by a broad range of UV–vis absorbing drugs, chromophoric probes, fluorescently labeled biomolecules, and nanoparticulate carriers with characteristic optical responses. In these cases, the time-resolved spectral signal remains a reliable surrogate for local concentration, enabling the extraction of trajectory-based dynamical descriptors without redesigning the analytical model. This suggests that the approach introduced here can be extended to chemically diverse systems, provided that their spectral evolution reflects the underlying transport dynamics in a reproducible manner.

## 5 Conclusions

In this work, we present a data-driven strategy to investigate drug release dynamics from bioprinted hydrogel patches, with direct relevance to localized delivery and biomedical applications. Using alginate-based soft matrices fabricated by extrusion bioprinting and loaded with indocyanine green as a spectroscopic probe, we show that time-resolved UV–vis absorbance measurements, analyzed through singular value decomposition and spectral trajectory mapping, provide a compact and physically meaningful description of the release process. This approach enables the extraction of characteristic release time scales directly from experimental data, without relying on predefined diffusion or kinetic models, offering a robust framework for the quantitative characterization of complex release phenomena in bioprinted platforms. Focusing on alginate-gelatin and calcium-cross-linked alginate-PVA patches as representative model systems, we demonstrate that singular value decomposition of time-resolved UV–vis absorbance spectra provides direct access to the intrinsic dynamical fingerprints of coupled swelling, transport, and release processes, without invoking a priori mechanistic assumptions. In both systems, the experimental spectra exhibit a characteristic accumulation behavior in the surrounding aqueous bath, consistent with an initially rapid appearance of the dye followed by progressive saturation. This evolution is quantitatively captured by the singular value spectrum, which displays a clear dimensional hierarchy. The dominance of the first singular components indicates that the release dynamics are effectively governed by a small number of collective degrees of freedom, despite the underlying complexity of transport within the hydrogel matrices.

Analysis of the leading temporal modes reveals distinct kinetic regimes, including an early transient phase associated with rapid local appearance of indocyanine green and a late-time stabilization phase corresponding to the equilibration of apparent local release availability at the measurement point. The extraction of characteristic times *t*_50_ and *t*_90_ from the reduced SVD representation provides robust and reproducible quantitative descriptors of these regimes. The remarkably small uncertainties obtained via bootstrap resampling^25,26^ reflect the smoothness and internal consistency of the dominant dynamical modes, as well as the strong signal-to-noise separation achieved through dimensional reduction.^27^

Beyond these temporal markers, the reduced spectral trajectories in the (*V*_1_, *V*_2_) plane offer a compact and physically transparent visualization of the release process. Their morphology encodes additional dynamical descriptors—such as the burst index, swelling index, and network resistance index—that complement *t*_50_ and *t*_90_ and provide a more complete characterization of the release dynamics. The emergence of broad maxima, inflection regions, or multiregime pathways in the spectral trajectories reflects the interplay between surface-mediated transport, swelling kinetics, and network relaxation, and highlights the sensitivity of the method to structural differences between hydrogel formulations. Importantly, the present results show that classical analytical models such as Higuchi or Korsmeyer-Peppas cannot be applied globally to these systems. Their underlying assumptions—rigid matrices, constant diffusivity, and purely Fickian transport—are incompatible with the swelling, time-dependent structural relaxation, and convective contributions observed in the bioprinted hydrogels studied here. In contrast, the SVD-based approach and the associated spectral trajectories provide a model-independent and physically grounded framework that allows the data to reveal the underlying regimes without imposing restrictive kinetic assumptions. Beyond the specific systems investigated here, the proposed methodology is general and transferable. It can be readily applied to a wide range of soft materials, hydrogel-based delivery platforms, and spectroscopically active drugs or probes. By shifting the focus from fitting predefined mechanistic models to extracting intrinsic dynamical structures directly from experimental data, this approach opens new avenues for the quantitative characterization of release kinetics and apparent local bioavailability in complex supramolecular environments. We anticipate that spectral trajectory analysis will provide a valuable tool for the rational design, optimization, and comparative assessment of advanced drug delivery systems, bridging spectroscopy, transport physics, and soft matter chemistry within a unified, model-independent framework. The two bioprinted patches investigated here, non-cross-linked alginate-gelatin and ionically cross-linked alginate-PVA, serve as representative examples of soft, weakly structured matrices and compact, relaxation-controlled networks. Their contrasting behaviors demonstrate the versatility of the proposed SVD-based trajectory analysis, which remains effective across fundamentally different transport regimes. By extracting intrinsic dynamical descriptors directly from experimental data, the method overcomes the limitations of classical analytical models and provides a unified framework for comparing materials that would otherwise require distinct mechanistic treatments. This trajectory-based perspective offers a powerful advantage: it condenses complex swelling, transport, and network-relaxation phenomena into a low-dimensional, physically interpretable representation, enabling robust and transferable characterization of drug release in advanced hydrogel systems.
